# Mercury concentrations in Seaside Sparrows and Marsh Rice Rats differ across the Mississippi River Estuary

**DOI:** 10.1007/s10646-024-02789-1

**Published:** 2024-07-24

**Authors:** Andrea Bonisoli-Alquati, Allyson K. Jackson, Collin A. Eagles-Smith, Sydney Moyo, Anna A. Pérez-Umphrey, Michael J. Polito, Allison M. Snider, S. Tyler Williams, Stefan Woltmann, Philip C. Stouffer, Sabrina S. Taylor

**Affiliations:** 1 Department of Biological Sciences, California State Polytechnic University, Pomona, Pomona, CA 91768 USA; 2 Purchase College SUNY, Department of Environmental Studies, Purchase, NY 10577 USA; 3 U.S. Geological Survey, Forest and Rangeland Ecosystem Science Center, 3200 SW Jefferson Way, Corvallis, OR 97331 USA; 4 Louisiana State University, Department of Biological Sciences, Baton Rouge, LA 70803 USA; 5 School of Renewable Natural Resources, Louisiana State University and LSU AgCenter, Baton Rouge, LA 70803 USA; 6 Louisiana State University, Department of Oceanography and Coastal Sciences, Baton Rouge, LA 70803 USA; 7 Center of Excellence for Field Biology, and Department of Biology, Austin Peay State University, Clarksville, TN 37040 USA

**Keywords:** Biomagnification, BP oil spill, Mercury, Saltmarsh, Trophic web

## Abstract

Mercury (Hg) concentrations and their associated toxicological effects in terrestrial ecosystems of the Gulf of Mexico are largely unknown. Compounding this uncertainty, a large input of organic matter from the 2010 Deepwater Horizon oil spill may have altered Hg cycling and bioaccumulation dynamics. To test this idea, we quantified blood concentrations of total mercury (THg) in Seaside Sparrows (*Ammospiza maritima*) and Marsh Rice Rats (*Oryzomys palustris*) in marshes west and east of the Mississippi River in 2015 and 2016. We also tested for a difference in THg concentrations between oiled and non-oiled sites. To address the potential confounding effect of diet variation on Hg transfer, we used stable nitrogen (δ^15^N) and carbon (δ^13^C) isotope values as proxies of trophic position and the source of primary production, respectively. Our results revealed that five to six years after the spill, THg concentrations were not higher in sites oiled by the spill compared to non-oiled sites. In both species, THg was higher at sites east of the Mississippi River compared to control and oiled sites, located west. In Seaside Sparrows but not in Marsh Rice Rats, THg increased with δ^15^N values, suggesting Hg trophic biomagnification. Overall, even in sites with the most elevated THg, concentrations were generally low. In Seaside Sparrows, THg concentrations were also lower than previously reported in this and other closely related passerines, with only 7% of tested birds exceeding the lowest observed effect concentration associated with toxic effects across bird species (0.2 µg/g ww**)**. The factors associated with geographic heterogeneity in Hg exposure remain uncertain. Clarification could inform risk assessment and future restoration and management actions in a region facing vast anthropogenic changes.

## Introduction

Mercury (Hg) is a toxic metal that has increased globally due to human activities, including burning of fossil fuels, especially coal, and mining, particularly artisanal and small-scale gold mining (Nriagu and Pacyna 1988; Outridge et al. 2018). Its methylated form, methylmercury (MeHg), poses health threats to humans and wildlife due to its potential to biomagnify through food webs, increasing its immunotoxicity, neurotoxicity, and endocrine disruptive activity (Ackerman et al. 2016; Eagles-Smith et al. 2018).

Drivers of geographic and topographic variation in Hg deposition, MeHg production and source apportionment, and exposure to wildlife are complex and vary across landscapes (Lindberg et al. 2007). The Gulf of Mexico is no exception, with drivers of Hg variation within the Gulf not well understood (Harris et al. 2012). Large uncertainties also exist with respect to the conversion of Hg to MeHg and its bioaccumulation in Gulf ecosystems, from marine to brackish to freshwater. At a local scale, a salinity gradient in Barataria Bay in the northern Gulf of Mexico is inversely associated with Hg concentrations in fish (Fry and Chumchal 2012). Sustained diversion of the Mississippi River flow into Breton Sound, east of the main river channel, was correlated with low salinity and the highest Hg concentrations found in the region (Fry and Chumchal 2012). Yet, to date, MeHg transfer to terrestrial wildlife and subsequent bioaccumulation have not been characterized on the two sides of the Mississippi River.

Organic matter input from the 2010 Deepwater Horizon (DWH) oil spill had the potential to alter MeHg production and subsequent food web transfer, although oil from the spill had a low concentration of Hg, making direct contributions unlikely (Wilhelm et al. 2007). Even without direct Hg input, three mechanisms could have influenced Hg methylation and transfer. First, the aromatic-rich organic input from the DWH oil spill may have contributed dissolved aromatic-rich organic matter to the system, either promoting or inhibiting MeHg production and bioaccumulation, depending on the level of oiling, characteristics of the organic matter, and the microbial groups present (Ravichandran 2004; Lavoie et al. 2019). Second, organic matter of petrogenic origin might have created reducing conditions favorable to MeHg production in coastal sediments, as proposed for deep-sea sediments (Hastings et al. 2016). A third mechanism is also possible if oil from the spill favored sulfate-reducing bacteria and other methylators. Several lines of evidence support this third mechanism. For instance, oil-derived increases in dissolved organic carbon (DOC) and bacterial enzymatic activity indicative of heterotrophic oil degradation were detected in surface waters from the DWH oil spill site (Ziervogel et al. 2012). Moreover, shifts in bacterial communities occurred in the oiled marshes, although this seemed to have happened indirectly due to changes in the vegetation rather than to direct oil exposure (Engel et al. 2017).

We know of no analyses of Hg concentrations in terrestrial populations following the spill or in relation to trophic levels. A recent study found no difference in MeHg concentrations in post-oil-spill eastern oysters (*Crassostrea virginica*) collected from Barataria Bay, LA, in 2010 compared with ones collected before the spill from 1986 to 2007 (Lamb et al. 2022). A study of tilefish (*Lopholatilus chamaleonticeps*) found that the input of suspended particles by the Mississippi River reduced Hg bioavailability in local food webs, an effect locally exacerbated by oil contamination from the DWH oil spill (Perrot et al. 2018).

We evaluated Hg concentrations in whole blood of adult Seaside Sparrows (*Ammospiza maritima*) and Marsh Rice Rats (*Oryzomys palustris*) on the east and west sides of the Mississippi River in 2015 and 2016. Due to their abundance and site fidelity, these two species are representative of vertebrate taxa that rely on near-shore environments affected by the spill (Bergeon-Burns et al. 2014). To test for relationships between trophic ecology and Hg concentrations we used nitrogen (δ^15^N) and carbon (δ^13^C) stable isotope values of whole blood in both species. We used δ^15^N values as a proxy of the trophic position of each organism, as previously documented for wildlife (Cristol et al. 2008; Lavoie et al. 2013; Lamb et al. 2022), and δ^13^C values to characterize foraging habitat (Kelly 2000). In both species, we tested whether oiling as a result of the DWH oil spill was associated with higher blood concentrations of Hg.

We predicted higher Hg concentrations in: (I) organisms from sites east of the Mississippi River because of the lower salinity, and (II) oiled sites compared to the non-oiled sites, due to aromatic-rich DOC from oil facilitating MeHg production. We also predicted that: (III) Hg concentrations would increase with trophic position, as indicated by δ^15^N values, and that this would be most pronounced east of the Mississippi.

## Methods

### Sample collection from Seaside Sparrows and Marsh Rice Rats

**Fig. 1:**
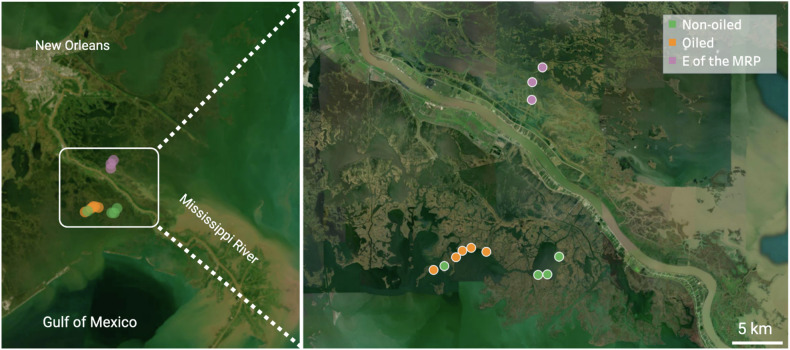
Map of the sampling sites, colored according to their oiling history (orange: oiled; green: non-oiled (i.e., control); purple: reference sites east of the Mississippi River Plume (MRP). Basemap from: openstreetmap.org/copyright

The 12 study sites were in Bay Sansbois, Bay Batiste, and Bay Jimmy, within the broader Barataria Bay, and in the Breton Sound Estuary, east of the Mississippi River (Fig. 1). Sites classified within different categories of oiling (see *Site classification and sediment analyses* below) were located at a minimum of 1 km away from one another. The sites were ecologically similar, with vegetation predominantly of Black Needlerush (*Juncus roemerianus*), Smooth Cordgrass (*Sporobolus alterniflorus*), and Saltgrass (*Distichlis spicata*).

In April–June 2015 and 2016 we sampled blood from 154 adult Seaside Sparrows and 80 Marsh Rice Rats (permits: USGS BBL 22648; USFWS MB095918-0; LDWF LNHP-15-033, LNHP-16-048, LNHP-15-039, LNHP-16-056). Handling and sampling methods were approved by the Institutional Animal Care and Use Committee of the Louisiana State University AgCenter (protocol numbers: A2013-09 (rats 2013–2015), A2016-06 (rats 2016–2018), and A2015-04 (birds 2015–2017)). The captured birds and rats were part of a broader effort (2011–2018) to investigate exposure and subsequent effects of the Deepwater Horizon oil spill on various aspects of the genetics, physiology, behavior, and ecology of these two species (Bergeon-Burns et al. 2014; Bonisoli-Alquati et al. 2016; Olin et al. 2017; Perez-Umphrey et al. 2018; Bonisoli-Alquati et al. 2020; Moyo et al. 2021; Snider et al. 2022a).

Seaside Sparrows are year-round residents of Louisiana salt marshes. They have been used as bioindicators of Hg exposure to avifauna in salt marshes because of their high habitat specificity (Warner et al. 2010). Seaside Sparrows are omnivorous, and feed by gleaning at the boundary between the terrestrial and aquatic food webs (Snider et al. 2022a, b), which increases their likelihood of exposure to MeHg (Cristol et al. 2008). Adult Seaside Sparrows were caught either via targeted mist-netting at their nest, or by non-targeted mist-netting (mesh size: 34 mm). Upon capture, birds were weighed, sexed by the presence of a cloacal protuberance (male) or a brood patch (female), and banded with an aluminum leg band (US Geological Survey). Blood was collected into 70 µL heparinized capillary tubes via puncturing of the brachial vein. The tubes were sealed at both ends and stored on ice in the field for a maximum of 10 h, and then stored at –20 °C.

Marsh Rice Rats are semi-aquatic cricetid rodents that inhabit salt marshes throughout the year, feeding on a variety of aquatic and terrestrial invertebrates, berries and grains (Kruchek 2004). They also feed on Seaside Sparrow eggs (Hart et al. 2021). We trapped Marsh Rice Rats using Sherman live traps (H. B. Sherman Traps, Inc., Tallahassee, FL) during two sessions on each site each year. A total of 42 traps were set along three lines. The lines ran parallel to the coastline, starting 5 m inland, with each line 15 m farther inland. Along each line, traps were placed 10 m apart. We typically set traps in the late afternoon, and checked them the next morning, for three consecutive days, at two locations at a time. Traps were attached to polystyrene platforms to keep them afloat during high water. Each trap had a plastic roof to prevent direct sunlight and overheating. Traps were baited with oats and peanut butter. Estimated isotopic turnover times in blood of 19–22 days based on other species of small rodents (Miller et al. 2008) confirms no effect of any consumed bait on diet reconstruction or estimates of stable isotope values and trophic position. For each animal, we recorded sex, mass, and age (adults if >30 g, juveniles if <30 g). We collected blood (max. 50 µL) from adults via retro-orbital bleeding, after sedation using isoflurane. We also attached 1 cm ear tags (National Band and Tag Co., Newport, KY) on both ears. After the procedure, rats were released at their site of capture. Mark-recapture analyses are available in companion papers (Hart et al. 2021; Pérez‐Umphrey et al. 2022). Juvenile rats were excluded from the analyses in this paper to eliminate the confounding effects of age structure and age-related diet specialization in estimating Hg variation across sites.

### Site classification and sediment analyses

Sites west of the Mississippi River were classified as oiled or non-oiled (control) based on Shoreline Cleanup and Assessment Technique (SCAT) survey maps (Michel et al. 2013; Nixon et al. 2016). As a more proximate measure of potential exposure to DWH oil, we also quantified the hydrocarbon content of surface sediment samples (i.e., top ~10 cm) from each site (n = 1–7 samples per year, per site). Hydrocarbons were quantified with gas chromatography/mass spectrometry in selective ion monitoring mode (GC/MS-SIM), as previously described (Turner et al. 2014). Briefly, this approach quantified C_10_ to C_35_ normal alkanes, plus the isoprenoid hydrocarbons, pristane and phytane. It also quantified 2- to 6-ringed parent polycyclic aromatic hydrocarbons (PAHs) and corresponding C1 to C3 or C4 alkyl homologs (Turner et al. 2014). According to the criteria described by Turner et al. (2014), we also conducted source oil identification to confirm that the oil residues in the collected sediment samples were from the DWH oil spill. This qualitative approach analyzed the ratio patterns of triterpanes (hopanes), steranes and triaromatic steroids, comparing patterns of each sediment sample to known patterns of DWH (i.e., MC252) source oil.

Sediment concentrations of PAHs served as proxies of oil exposure in each site and year, as they remained 10 times higher than background contamination up until 2018, and likely longer (Turner et al. 2019; McClenachan and Turner 2023). Due to the small body size of the study species, the analyses of PAHs exposure from tissue concentrations would have required the sacrifice of individual animals. While some individuals were sacrificed to address related questions about exposure and response to oil (Perez-Umphrey et al. 2018; Bonisoli-Alquati et al. 2020), the remainder were essential to clarifying the effects of oiling of marshes on the reproductive success and population dynamics of these vertebrate species. Considerations about and permits for ethical use of animals in research did not allow for the sacrifice of a number of individual animals as large as the sample size reported here.

### Mercury (THg) analyses

In passerine birds, blood Hg concentrations reliably indicate recent dietary exposure to Hg (Evers et al. 2005). Typically, 95–99% of blood Hg concentration is MeHg (Rimmer et al. 2005). Thus, quantifying total blood Hg provides a reliable proxy of exposure to mercury’s most toxic, methylated form. Because Seaside Sparrows show high site fidelity (Greenlaw et al. 2022), the measured blood THg concentrations should thus mirror the fine-scale availability of MeHg. We quantified total Hg (THg) according to EPA method 7473 (U.S. Environmental Protection Agency 2007). For measuring THg we used a Milestone tri-cell DMA-80 Direct Hg Analyzer (Milestone, Shelton, Connecticut USA). The method uses combustion and gold amalgamation coupled with cold vapor atomic absorption spectrometry. Blood THg concentrations are expressed in ppm wet weight (ww). Certified reference material (dogfish muscle tissue, DORM-4, National Research Council of Canada, Ottawa Canada), calibration verification (liquid standards), air blanks, and boat blanks were included in each run. Total mercury QA/QC included recoveries of 102.6% (SD = 2.7%, N = 24) for certified reference material and 95.9% (SD = 6.6%, N = 12) for calibration verification.

### Stable isotopes analyses

We used δ^15^N values as a proxy of trophic position and δ^13^C values as a proxy of aquatic vs. terrestrial resource pathways. Consumers tend to retain the heavier nitrogen isotope ^15^N in their tissues compared to the lighter isotope ^14^N (Kelly 2000). Consequently, ^15^N becomes enriched in food webs with each trophic step, typically as a *ca*. 3‰ change per trophic level (Post 2002). This happens particularly in aquatic food webs compared to purely terrestrial ones (Kelly 2000; Post 2002). We measured δ^13^C values to characterize foraging habitat, with enrichment in ^13^C as a reliable signature of the source of primary production across a terrestrial to aquatic food web gradient (Kelly 2000). Higher δ^13^C values are typical of plants that use the C_4_ rather than C_3_ photosynthesis pathway (Kelly 2000). In consumers like Marsh Rice Rats and Seaside Sparrows, higher δ^13^C values indicate a higher proportion of carbon, and thus of overall resources, derived from C_4_ marsh grasses vs. aquatic sources such as algae (Post 2002; Marshall et al. 2008; Moyo et al. 2021).

Isotopic turnover in avian and mammalian tissues have an allometric relationship with body size (Zanden et al. 2015). For example, turnover of whole blood δ^13^C and δ^15^N values in whole blood has a half-life of 5–11 days for passerines (Hobson and Bairlein 2003) and 19–22 days for small rodents (Miller et al. 2008). Therefore, stable isotope values for Seaside Sparrow and Marsh Rice Rat whole blood should reflect diets and foraging habitat over the week to month prior to sampling. Blood samples from both species were kept on ice in the field, then transferred to a –20 °C freezer. Before stable isotope analyses, blood samples were freeze-dried, pulverized and transferred into tin capsules, where they were weighed before combustion. Samples were flash-combusted using a Costech ECS4010 Elemental Analyzer and analyzed for δ^13^C and δ^15^N values via a Thermo Scientific XP Ratio Mass Spectrometer. Stable isotope ratios are expressed using the standard δ notation in parts per mil (‰) deviations from standards, according to the following equation:

$$\delta {\rm{X}}=[({{\rm{R}}}_{{\rm{sample}}}/{{\rm{R}}}_{{\rm{standard}}})-1]$$

where X is ^13^C or ^15^N and R is the corresponding ratio ^13^C /^12^C or ^15^N /^14^N. The R_standard_ values were the Vienna PeeDee Belemnite (VPDB) for δ^13^C and atmospheric N_2_ for δ^15^N.

Raw δ values were normalized using low and high glutamic acid reference materials [i.e. USGS40 (δ^13^C = –26.4‰, δ^15^N = –4.5‰) and USGS41 (δ^13^C = 37.6‰, δ^15^N = 47.6‰)]. The analytical precision, based on standard deviations of repeated reference materials, were 0.1‰ and 0.2‰ for δ^13^C and δ^15^N values, respectively.

### Statistical analyses

To analyze THg concentrations in each species we used general linear mixed models (estimated using ML) with year and oiling (a three-level factor for non-oiled, oiled, and east of the Mississippi) as fixed effects, their interaction, and sediment concentration of PAHs (as a covariate), as implemented in the *lme4* package (Bates et al. 2015) in R version 4.3.1 (2023-06-16) (R Core Team 2023). A subset of Seaside Sparrows was captured multiple times in the same year (N = 12) or between subsequent years (N = 8). To account for the non-independence of those observations, we also included sampling site and individual identity, nested within sampling site, as random intercepts in models of blood THg concentrations in Seaside Sparrows. Conversely, only one Marsh Rice Rat was recaptured in the same year. Therefore, models of blood THg concentrations in Marsh Rice Rats only included sampling site as a random intercept. The statistical significance of the random effects in explaining variation in THg concentrations was tested using log-likelihood ratio tests. Standardized parameters were obtained by fitting the models on a standardized version of the dataset. 95% Confidence Intervals (CIs) and *p* values were computed using a Wald *t*-distribution approximation.

To test hypotheses regarding the potential enduring role of oiling and its subsequent mobilization and transport, we compared different statistical models by using an information theoretic approach. We compared Akaike Information Criterion corrected for small sample size (AICc; Burnham and Anderson 2002) scores in models with different combinations of factors and covariates, including sediment concentration of PAHs, oiling status, and year of collection (as factors). A model of oiling status alone tested the idea that Hg concentrations were directly influenced by oil input from the oil spill and its associated ecological effects. A separate model of sediment PAHs alone tested whether THg concentrations are instead best explained by more recent exposure to oil. The model that included both asked whether THg concentrations are best explained by both initial oiling and recent concentrations. A model of year effect alone tested whether interannual ecological variation best explains THg concentrations, rather than the legacy and contemporary effects of oiling. Finally, a model including all three predictors was run to test whether the best fit to the observed data was from a model accounting for legacy, recent, and interannual effects. Multiple linear regressions were run on standardized variables (mean = 0, SD = 1), to remove influences on effects due to scale differences in the different variables. We generated a full submodel set (including the null model) from the global model by using the *dredge* function implemented in the *MuMIn* package (Bartoń 2024). Residuals from the models were inspected for normality using the Shapiro–Wilk normality tests in the package *lmerTest* (Kuznetsova et al. 2017). Unless noted otherwise, THg concentrations were log-transformed to attain normality and are reported as geometric means to avoid biases due to individuals with extreme values.

The relationships between THg concentrations and C and N stable isotope values were tested using linear mixed models (estimated using ML), separately for each species and each stable isotope. The models included oiling history and the interaction between oiling history and stable isotope values (as a covariate), allowing for the relationship between THg and each stable isotope to vary depending on oiling history. When not statistically significant, the interaction was removed from the models, and the main effects of oiling and each stable isotope were tested. The models for Seaside Sparrows included sampling site and individual identity (nested within site) as random effects. In Marsh Rice Rats, the models only included the sampling site as a random effect, as no individual was included twice in the analyses.

To test for consistency in estimates of THg concentrations and stable isotopes, we fitted linear models (estimated using ordinary least squares (OLS) regression) to predict THg and stable isotopes as a function of individual identity. Of the birds with multiple estimates of THg, 13 also had multiple estimates of stable isotopes, enabling the repeatability of those estimates to be calculated. Because no individual was sampled at different sites, the sampling site was not included (as a random effect). Conversely, THg exposure for the recaptured Marsh Rice Rat was estimated by averaging the two measurements. Measurements of δ^15^N and δ^13^C values at second capture for this rat were not used to avoid the confounding effect of feeding on the bait at first capture.

## Results

### Total mercury (THg) concentrations in Seaside Sparrows

**Fig. 2:**
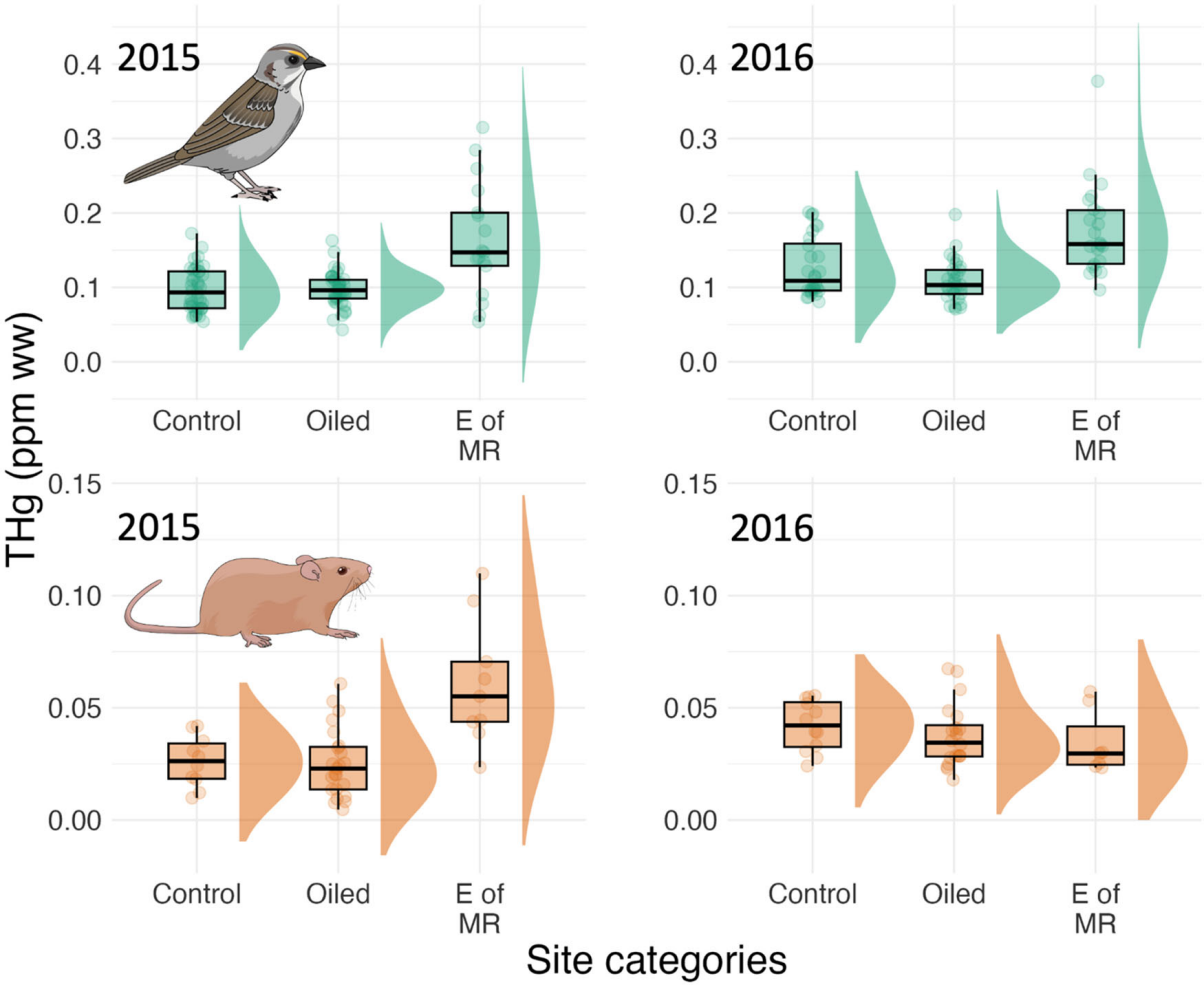
Boxplots and violin plots of total mercury (THg) concentrations in the blood of Seaside Sparrows and Marsh Rice Rats in 2015 and 2016, across site categories, including control sites (west of the Mississippi River), sites oiled by the Deepwater Horizon oil spill (also west of the Mississippi River), and sites east of the Mississippi River (E of MR). Note that the scale for THg (in parts per million (ppm) per unit wet weight (ww), or µg/g) is different in the two species, reflecting higher total concentrations in Seaside Sparrows than Marsh Rice Rats

Akaike’s Information Criterion scores for small sample sizes (AICc) indicated that for Seaside Sparrows the most informative model included oiling history and sampling year (Table 1). A model including sediment concentrations of PAHs (as a covariate), in addition to oiling history and sampling year (as a factor) ranked similarly (ΔAICc < 2; Table 1). The most informative model revealed extensive variation in THg among oiled sites, control sites, and sites east of the Mississippi River (*F*_2,10.47_ = 11.59, *p* = 0.002, n = 175; Fig. 2). Total mercury concentrations were similar between oiled sites and non-oiled sites (*non-oiled sites*: geometric mean THg concentrations = 0.103 µg/g (1.39 geometric SD), n = 67; *oiled sites*: geometric mean = 0.099 µg/g (1.29 geometric SD), n = 69; *t* = 0.365; *p* = 0.7244; Fig. 2). Seaside Sparrows from sites east of the Mississippi River had higher THg concentrations (geometric mean = 0.158 µg/g (1.51 geometric SD), n = 39) than birds from either the non-oiled sites (*t* = −3.44, *p* = 0.009) or the oiled sites (*t* = –3.85, *p* = 0.003). Overall, birds from sites west of the Mississippi River (both oiled and non-oiled) had circulating THg concentrations (geometric mean: 0.101 ppm (1.34 geometric SD)) that were 36% lower than those of birds east of the Mississippi River (*t* = 4.31, *p* = 0.002). Across sites, THg concentrations were higher in 2016 than in 2015 (*F*_1,169.9_ = 13.27, *p* = 0.0004). There was statistically significant variation in THg among sampling sites, regardless of initial oiling (likelihood ratio test (LRT) = 7.05, *p* = 0.008). Individual identity did not explain a significant amount of variation in THg (LRT = 0.45, *p* = 0.503).

**Table 1:** Seaside Sparrow THg concentration data of the ten best-performing general linear mixed models (with site and individual identity as random effects)

*Model*	*df*	*log* *likelihood*	*AICc*	*ΔAICc*	*w*_*i*_
**Year** **+** **Oiling**	**7**	**107.83**	**–201.0**	**0.00**	**0.588**
**Year** **+** **Oiling** + **[PAHs]**	**8**	**107.97**	**–199.1**	**1.92**	**0.225**
Year + Oiling + Year × Oiling	9	108.44	–197.8	3.46	0.119
Year + Oiling + Year × Oiling + [PAHs]	10	108.73	–196.1	4.87	0.052
Year	5	101.44	–192.5	8.47	0.009
Year + [PAHs]	6	101.44	–190.4	10.61	0.003
Oiling	6	101.44	–190.4	10.61	0.003
Oiling + [PAHs]	7	101.88	–189.1	11.89	0.002
*Intercept only*	4	94.83	–181.4	19.57	0.000
[PAHs]	5	95.00	–179.6	21.34	0.000

The models test various hypotheses about factors and covariates explaining inter-individual variation in THg

AICc are Akaike’s Information Criterion scores adjusted for small sample sizes (Burnham and Anderson 2002. ΔAICc is the difference between the AICc score of a given model and the lowest AICc of all models (i.e., the model including the effect of oiling history and year, in this case). *w*_*i*_ is the Akaike’s weight of each model. The best models are in bold. [PAHs] stands for sediment concentrations of polycyclic aromatic hydrocarbons

### Total mercury (THg) concentrations in Marsh Rice Rats

For Marsh Rice Rats, AICc scores indicated that the most informative model included oiling history, sampling year, their interaction, and sediment concentrations of PAHs (as a covariate; Table 2). Two models that didn’t include either the interaction between oiling history and sampling year or sediment concentrations of PAHs ranked similarly (ΔAICc <2; Table 2). Among Marsh Rice Rats, variation in THg across oiling categories depended on sampling year (*interaction oiling x year*: *F*_2,75.65.02_ = 7.61, *p* = 0.001; Fig. 2). In 2015, rats from sites east of the Mississippi River had THg concentrations (geometric mean = 0.055 µg/g (1.61 geometric SD), n = 9) higher than the rats from oiled sites (geometric mean = 0.021 µg/g (1.96 geometric SD), n = 23; *t* = –3.36, *p* = 0.006) or non-oiled sites (geometric mean = 0.024 µg/g (1.64 geometric SD), n = 10; *t* = –2.59, *p* = 0.036). THg concentrations did not differ between oiled and non-oiled sites (*t* = 0.58, *p* = 0.834). The pattern changed in 2016, when THg concentrations were similar across all groups of sites (sites east of the Mississippi River: geometric mean = 0.033 µg/g (1.45 geometric SD), n = 7; oiled sites: geometric mean = 0.035 µg/g (1.43 geometric SD), n = 20; control sites: geometric mean = 0.040 µg/g (1.33 geometric SD), n = 12). The overall difference between the two years was driven by an increase in THg in 2016 compared with 2015 in non-oiled sites (*t* = –2.52, *p* = 0.014) as well as in oiled sites (*t* = –2.304, *p* = 0.0238), and a non-statistically significant decrease in THg in sites east of the Mississippi River (*t* = 1.16, *p* = 0.25). THg tended to decline with sediment concentrations of PAHs (std. beta = –0.28, 95% CI [−0.57, 0.01]; *t* = –1.90, *p* = 0.061).

**Table 2:** Comparison of the fit to Marsh Rice Rat THg concentration data of the ten best-performing general linear mixed models (with site as a random effect) explaining variation in total Hg (THg) concentrations in Marsh Rice Rats in 2015–2016

*Model*	*df*	*log* *likelihood*	*AICc*	*ΔAICc*	*w*_*i*_
**Year** **+** **Oiling** **+** **Year** **×** **Oiling** + **[PAHs]**	**9**	**14.93**	**–9.3**	**0.00**	**0.441**
**Year** **+** **Oiling** + **Year** **×** **Oiling**	**8**	**13.36**	**–8.7**	**0.59**	**0.328**
Year + Oiling + [PAHs]	**7**	**11.46**	**–7.4**	**1.93**	**0.168**
Oiling + [PAHs]	6	8.72	–4.3	5.02	0.036
Year + [PAHs]	5	6.57	–2.3	6.99	0.013
[PAHs]	4	4.78	–1.0	8.29	0.007
Year + Oiling	6	6.26	0.6	9.93	0.003
Year	4	3.86	0.8	10.11	0.003
*Intercept only*	3	1.33	3.6	12.97	0.001
Oiling	4	3.18	4.4	13.76	0.000

AICc are Akaike’s Information Criterion scores adjusted for small sample sizes. ΔAICc is the difference between the AICc score of a given model and the lowest AICc of all models (i.e., the model including oiling history, year, and their interaction, in this case). The best models are in bold. *w*_*i*_ is the Akaike’s weight of each model. [PAHs] stands for sediment concentrations of polycyclic aromatic hydrocarbons

### Hg and stable isotopes

Stable isotope values varied according to oiling history of the sites and sampling year (Supplementary Fig. S1). Also, δ^13^C and δ^15^N values were more strongly associated for Seaside Sparrows (linear regression: adj. *R*^*2*^ = 0.35, estimate (SE) = –1.143 (0.133), *t* = –8.621, *p* < 0.0001) than for Marsh Rice Rats (adj. *R*^*2*^ = 0.01, estimate (SE) = –0.2266 (0.3752), *t* = –0.604, *p* = 0.548; Supplementary Fig. S2b).

**Fig. 3:**
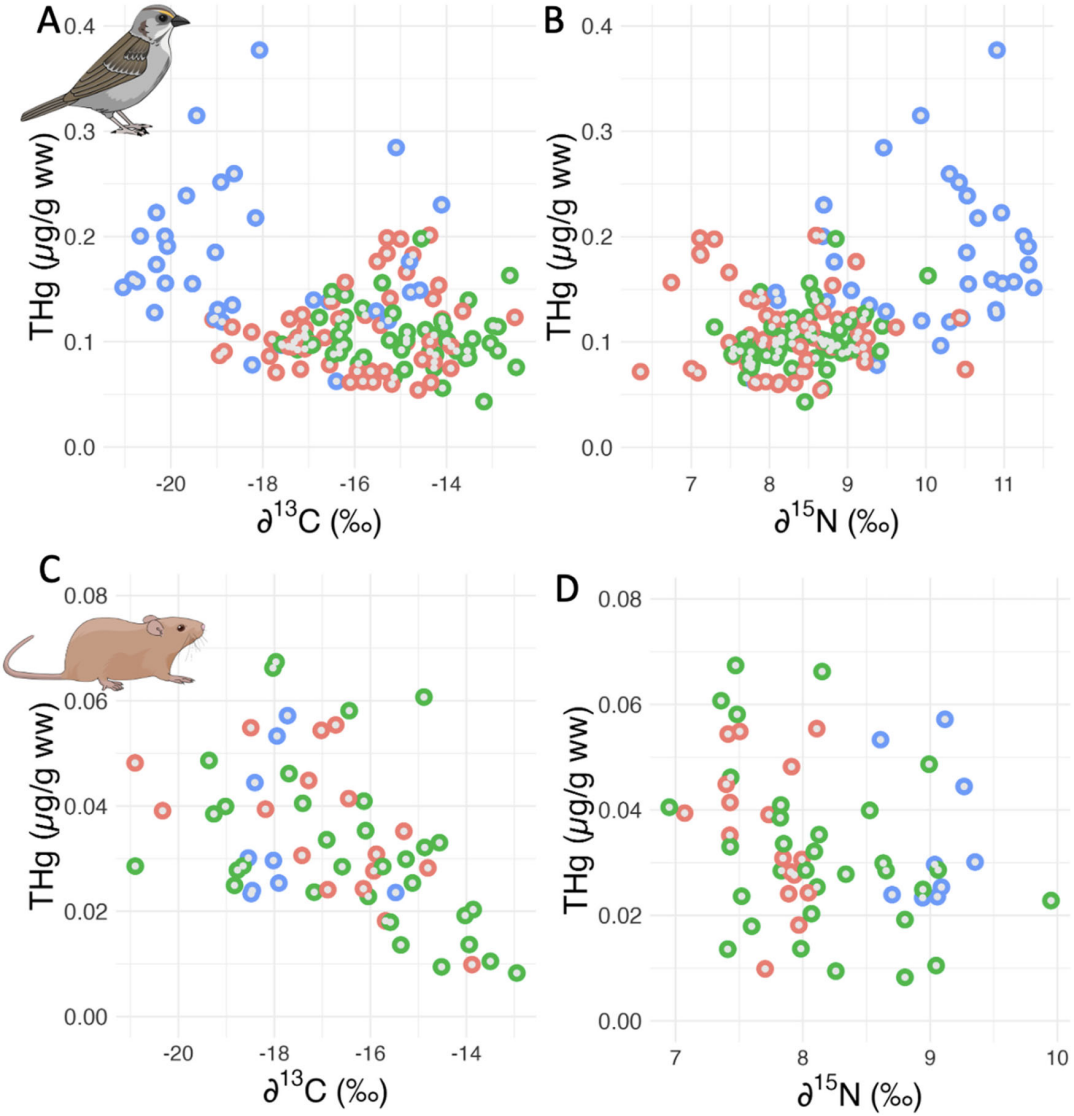
Total mercury (THg) concentrations in the blood of Seaside Sparrows (top) and Marsh Rice Rats (bottom) as a function of δ^13^C (a marker of the provenance of food items; **A**, **C**) and δ^15^N values (a marker of trophic level; **B**, **D**), across site categories, including non-oiled sites (red), sites oiled by the Deepwater Horizon oil spill (green), and sites east of the Mississippi River (blue). Note that the scale for THg (in µg/g wet weight (ww), or parts per million (ppm)) is different in the two species, consistent with higher total concentrations in Seaside Sparrows than Marsh Rice Rats

In both Seaside Sparrows and Marsh Rice Rats, THg decreased with δ^13^C values (Seaside Sparrows: *t* = –2.57, *p* = 0.011; std. beta = –0.25, 95% CI [–0.43, –0.06]; n = 138; Marsh Rice Rats: *t* = –−5.48, *p* < 0.001; std. beta = –0.57, 95% CI [–0.78, –0.36]; n = 58; Fig. 3). The relationship between blood THg concentrations and δ^15^N values in Seaside Sparrows depended on oiling history (*interaction oiling x δ*^*15*^*N*: *F*_2,131.27_ = 3.99; *p* = 0.021; n = 138; Fig. 3). In rats, THg concentrations decreased with δ^15^N values (*t* = –2.46; *p* = 0.017; std. beta = –0.39, 95% CI [–0.71, –0.07]; n = 58; Fig. 3). The effect of the sampling site was statistically significant in Seaside Sparrows models (*p* ≤ 0.001), but not for Marsh Rice Rats (*p* ≥ 0.1 in all cases). The random effect of bird identity was never statistically significant (*p* > 0.077 in all cases).

### Repeatability of Hg and stable isotopes

The effect of individual identity was statistically significant in predicting THg in the subset of Seaside Sparrows recaptured twice or more (*F*_14, 20_ = 2.48; *p* = 0.032), indicating within-individual consistency in Hg exposure. The model explained a substantial proportion of variance (adj. *R*^*2*^ = 0.38). The same was generally true for models of δ^13^C and δ^15^N values: the effect of individual identity was statistically significant in both (δ^13^C: *F*_13, 16_ = 4.68; *p* = 0.002; δ^15^N: *F*_13, 16_ = 5.35; *p* = 0.001), and explained a substantial proportion of variance (δ^13^C: adj*. R*^*2*^ = 0.62; δ^15^N: adj. *R*^*2*^ = 0.66).

## Discussion

We documented extensive variation in blood total mercury (THg) concentrations in two vertebrate species that reside in salt marshes on both sides of the Mississippi River. Mercury concentrations varied ~1.5 fold and ~2.6 fold between groups of sites in Seaside Sparrows and Marsh Rice Rats, respectively. Despite this variability, overall THg concentrations were fairly low in both species, with less than 10% of individuals exceeding concentrations associated with health impairment (Ackerman et al. 2016). Contrary to our predictions, THg concentrations did not differ between non-oiled sites and those oiled by the Deepwater Horizon oil spill. Thus, 5–6 years after the spill, there was no evidence that residual oiling altered marsh biogeochemistry or community composition in such a way that influenced Hg exposure in these species.

The lack of an apparent influence of oiling on Hg transfer and magnification is consistent with a recent study in Barataria Bay, the same general location as our study, that documented no differences in tissue Hg concentrations of oysters immediately after the Deepwater Horizon oil spill (Lamb et al. 2022). Evidence from Bottlenose Dolphins (*Tursiops truncatus*) collected as part of the Deepwater Horizon oil spill environmental assessment is also in line with our results (McCormack et al. 2020): exposure to contaminated waters did not affect skin Hg concentrations in Louisiana dolphins. In fact, the highest Hg concentrations were in dolphins from Florida waters (McCormack et al. 2020). The samples analyzed here were collected in 2015 and 2016 (i.e., 5 and 6 years post-spill), so caution should be applied in extrapolating these results to earlier years. Significant transport and redistribution of the spilled oil residues occurred throughout the years since the disaster, caused by extreme weather events like Hurricane Isaac (in 2012) and tropical storms (Diercks et al. 2021; Justić et al. 2022). Consistent with the remobilization, transport, and redistribution of oil residues, higher exposure to PAHs was documented by biomarkers in studies of marine (Romero et al. 2018; Morey et al. 2022) and terrestrial organisms (Perez-Umphrey et al. 2018), up to several years after the spill and away from the originally oiled sites. Still, when we used sediment concentrations of PAHs temporally concordant with bird and rat sampling as proxies of oil residue redistribution, the lack of a relationship between Hg concentrations and oiling remained. More accurate measures of exposure to PAHs (i.e., their tissue concentrations) could reveal a relationship between Hg concentrations and oiling. Those measures were not possible in all individuals due to the small body size of our study organisms and the broader context of studying their reproductive success (Hart et al. 2021) as well as population dynamics (Pérez‐Umphrey et al. 2022).

The environmental drivers of differences in Hg concentration across the Mississippi River, and their variation between years, remain to be clarified. Several factors affect net methylmercury (MeHg) production and bioavailability in aquatic environments (review in Ullrich et al. 2001), which could have contributed to variation in THg across sites. For example, the observed differences in THg could be due to the extensive, ten-fold difference in salinity between the two sides of the Mississippi near our field sites (Coastwide Reference Monitoring System (CRMS); https://www.lacoast.gov/crms). Previous studies of Seaside Sparrows in Delaware Bay have found that birds from sites further inland had higher Hg concentrations in their blood, which the authors interpreted as the result of higher freshwater Hg input and/or lower tidal circulation (Warner et al. 2010). However, circulating Hg concentrations in Swamp Sparrows (*Melospiza georgiana*) from Wisconsin wetlands did not differ between birds from acidic wetlands in the north and birds from the less-acidic swamps in the southern part of the state (Strom and Brady 2011).

Higher Hg concentrations east versus west of the Mississippi River have been documented in fish communities (Fry and Chumchal 2012) and bottlenose dolphins (McCormack et al. 2021), matching the pattern we found here in both our terrestrial species (although only in one of the two years for Marsh Rice Rats). Our data add to the known geographic differences in sediment and tissue concentrations of Hg among sectors of the Gulf of Mexico (Apeti et al. 2012; Lamb et al. 2022), whereas other studies failed to show any clear spatial structuring in THg concentrations (Cai et al. 2007). Overall, it remains unclear how generalizable differences in Hg tissue concentrations are, and whether Hg bioaccumulation in aquatic and terrestrial fauna is coupled across space and time.

Differences in foraging ecology may also be responsible for higher blood Hg concentrations in Seaside Sparrows from east of the Mississippi River compared to birds from the western side, as evidenced by corresponding spatial differences in THg and stable isotope values. In support of this concept, higher Hg concentrations in Marsh Rice Rats from east of the Mississippi occurred only in 2016, when their δ^15^N values were higher than those from the previous year or than Marsh Rice Rats from other sites. Alternatively, Seaside Sparrows on each side of the river may have been feeding at a similar trophic position, while their blood δ^15^N values differed due to higher food web baseline δ^15^N values east of the Mississippi. This would be consistent with the greater input of fresh water with higher δ^15^N particulate organic matter values and baseline Hg concentrations at sites east of the Mississippi (Wissel and Fry 2005; Fry and Chumchal 2012).

Still, Hg concentrations in both species were generally low to very low, even east of the Mississippi River. Only 13 of 175 birds (~7%) exceeded the lowest observed effect concentration (LOEC) of 0.2 µg/g ww, established by a systematic review and meta-analysis that compiled Hg concentrations and associated toxic effects across 225 bird species (Ackerman et al. 2016). As a reference, 66% of the almost 30,000 North American birds included in the meta-analysis had concentrations above that same LOEC (Ackerman et al. 2016; Eagles-Smith et al. 2016). None of the birds we sampled had blood THg concentrations that exceeded 0.7 μg g^–1^, a concentration known to cause a 10% reduction in probability of successful reproduction (Jackson et al. 2011). Here, THg concentrations in Seaside Sparrows were also lower than those reported from the Atlantic Coast and in the closely related Saltmarsh Sparrow (*A. caudacuta*) (Warner et al. 2010; Winder and Emslie 2011; Winder 2012; Sayers et al. 2021; Ruskin et al. 2022). However, our data are consistent with those reported for Nelson’s Sparrows (*A. nelsoni*) and Swamp Sparrows (Strom and Brady 2011; Ruskin et al. 2022). These geographic and interspecific differences are difficult to explain, with foraging behavior, dietary specializations, industrial input, and differences in toxicokinetics as plausible causes (Cristol and Evers 2020; Sayers et al. 2021). Our results are therefore consistent with recent evidence that blood concentrations vary greatly among wetlands, possibly exceeding the differences between wetlands and other ecosystems (Sayers et al. 2021).

Even with overall low Hg concentrations, there was extensive intraspecific variation, with up to five-fold differences within a site in a given year. Much of this variation among individuals remains unexplained, a situation not unique to our study. Breeding status at sampling, and variation in the degree of maternal offloading of Hg through eggs, could explain some of the unaccounted variation (Hitchcock et al. 2019), as monitoring of reproduction was not feasible for all individuals sampled. Age and the extensive intraspecific foraging and dietary specialization in this same population (Snider et al. 2022a) may also have contributed to differences in Hg exposure.

### Hg and stable isotopes

In a previous study on these same two species, we showed that trophic position (i.e., δ^15^N values) was similar in oiled and non-oiled sites, although trophic niche width was more variable across years at oiled sites (Moyo et al. 2021). δ^13^C values indicated that Seaside Sparrows relied more on terrestrial (i.e., emergent C4 vegetation) than aquatic (i.e., suspended particulate organic matter) basal resource pathways, while Marsh Rice Rats relied more on aquatic resources in two of three years (Moyo et al. 2021). Here, we showed that Hg concentrations in Seaside Sparrows increased with δ^15^N values, a potential indication of increasing concentrations with trophic position. This is consistent with results from a marked Hg biomagnification study across various trophic levels, from filter feeding bivalves to piscivorous birds, in Barataria Bay (Lamb et al. 2022). This result also confirms previous statistically significant yet weak correlations reported between δ^15^N values and blood and feather Hg concentrations in New England Saltmarsh, Seaside, and Nelson’s sparrows (Cristol et al. 2011; Winder et al. 2012). However, oiling history did influence this correlation. Among Marsh Rice Rats, δ^15^N values negatively predicted Hg concentrations, although it should be noted that the sample size was smaller and the range of variation in trophic level narrower in this species, limiting our inference. The lack of available stable isotope values as a food web baseline at these sites precluded us from explicitly calculating consumer trophic position (e.g., Brasso and Polito 2013) and quantification of biomagnification across sites (e.g., Lamb et al. 2022). Additional information on food webs would clarify the mechanisms influencing the observed differences in Hg concentrations and stable isotope values between the two sides of the Mississippi River.

In both species, Hg concentrations also covaried with the specific basal carbon pathways used, as estimated by δ^13^C values. Such an inverse correlation is difficult to interpret unequivocally. Algae have higher δ^13^C values than terrestrial plants due to differences in the pool of carbon and/or photosynthetic pathways used (Kelly 2000). A lower contribution of aquatic carbon to the diet of terrestrial species in sites east of the Mississippi River would be consistent with higher Hg methylation and transfer rates under the more riverine (i.e., freshwater) conditions found there. On the other hand, our findings conflict with previous research showing ^13^C enrichment in Breton Sound compared to Barataria Bay for POM (Wissel and Fry 2005) and fishes (Fry and Chumchal 2012). For Seaside Sparrows, δ^13^C values likely depended more on differences in relative use of C_4_ marsh grasses and C_3_ algal/POM carbon than on variation in POM (C_3_) values themselves (Moyo et al. 2021).

Irrespective of the Hg dietary source, our results have implications for the planning and operation of freshwater diversions. These diversions include the Mid-Barataria Sediment Diversion and Bonnet Carré Freshwater Diversion Projects, which recently began construction after decades of planning. These diversions are intended as sources of sediments to the estuary to mitigate wetland loss (Allison and Meselhe 2010; Nittrouer et al. 2012). These episodic river diversions, such as the mid-Barataria sediment diversion, will also reduce salinity in the Mississippi estuary (Ou et al. 2020). Opposition to these engineering interventions emphasizes their over-freshening and nutrient release, with unknown ecosystem consequences (Turner and Rabalais 1991; Turner et al. 2007; Day et al. 2017). If changing salinity also influences Hg cycling and bioavailability, increasing the influx of Mississippi waters to Barataria Bay could change MeHg exposure in fish and wildlife. Fish consumption is the primary Hg exposure route in humans (Sunderland 2007), and Gulf fisheries account for more than 16% of commercial and 41% of recreational landings in the United States (NOAA, 2011). This highlights the importance of understanding the factors influencing Hg exposure in biota in Gulf waters for human Hg intake. Our results suggest that changing salinity, as a result of diversion projects or other mechanisms, may have implications for the increase in MeHg transfer to terrestrial wildlife.

In summary, five to six years after oiling of the Louisiana salt marshes from the Deepwater Horizon oil spill, we did not detect a legacy of historical oiling or an effect of sediment concentrations of PAHs on blood THg concentrations of terrestrial vertebrates. THg concentrations were overall low, compatible with minor toxicological effects, and lower than in the same species or ecologically similar ones elsewhere (i.e., coastal sparrow species in North Atlantic tidal marshes). Still, our analyses revealed substantial individual and spatial variation in blood THg concentrations, particularly in wildlife on opposite sides of the Mississippi River.

## Supplementary Information

Supplementary Material
