# Supplementary material for: Mercury concentrations in Seaside Sparrows and Marsh Rice Rats differ across the Mississippi River Estuary
Source: Ecotoxicology. 2024 Jul 24;33(8):959–71. doi: 10.1007/s10646-024-02789-1 (PMC11399195; doi:10.1007/s10646-024-02789-1)
Supplement: Supplementary file 1 — Supplementary Material [file 10646_2024_2789_MOESM1_ESM.docx]

**Supplementary Material**


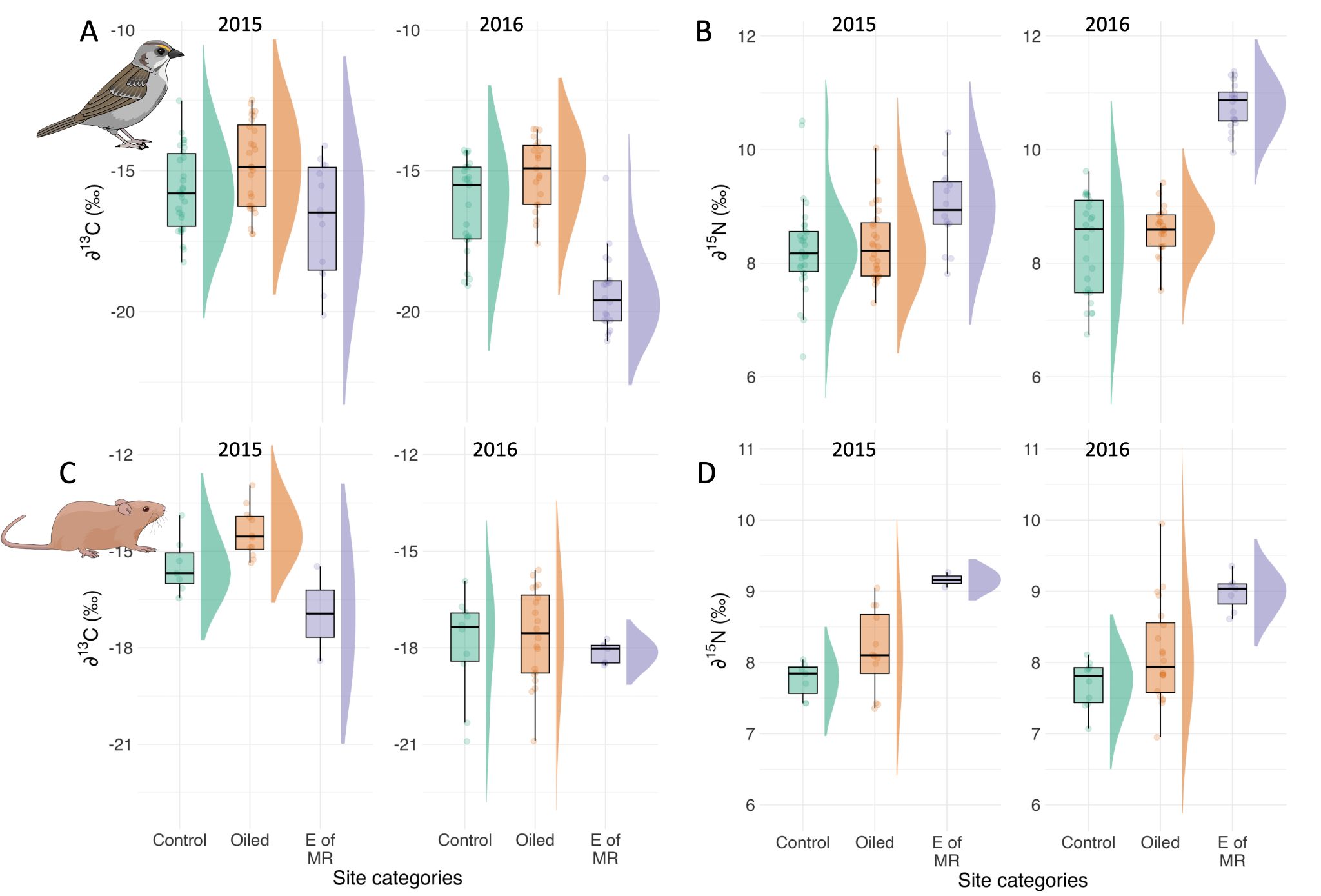


Figure S1. Distributions of carbon (∂^13^C; A-C) and nitrogen (∂^15^N; B-D) stable isotope values for Seaside Sparrows (A-B) and Marsh Rice Rats (C-D) in sites with different oiling histories (control, oiled, and sites east of the Mississippi River, well outside the initial impact of the oil spill). Data are shown separately by year, to illustrate interannual variation in stable isotopes values.


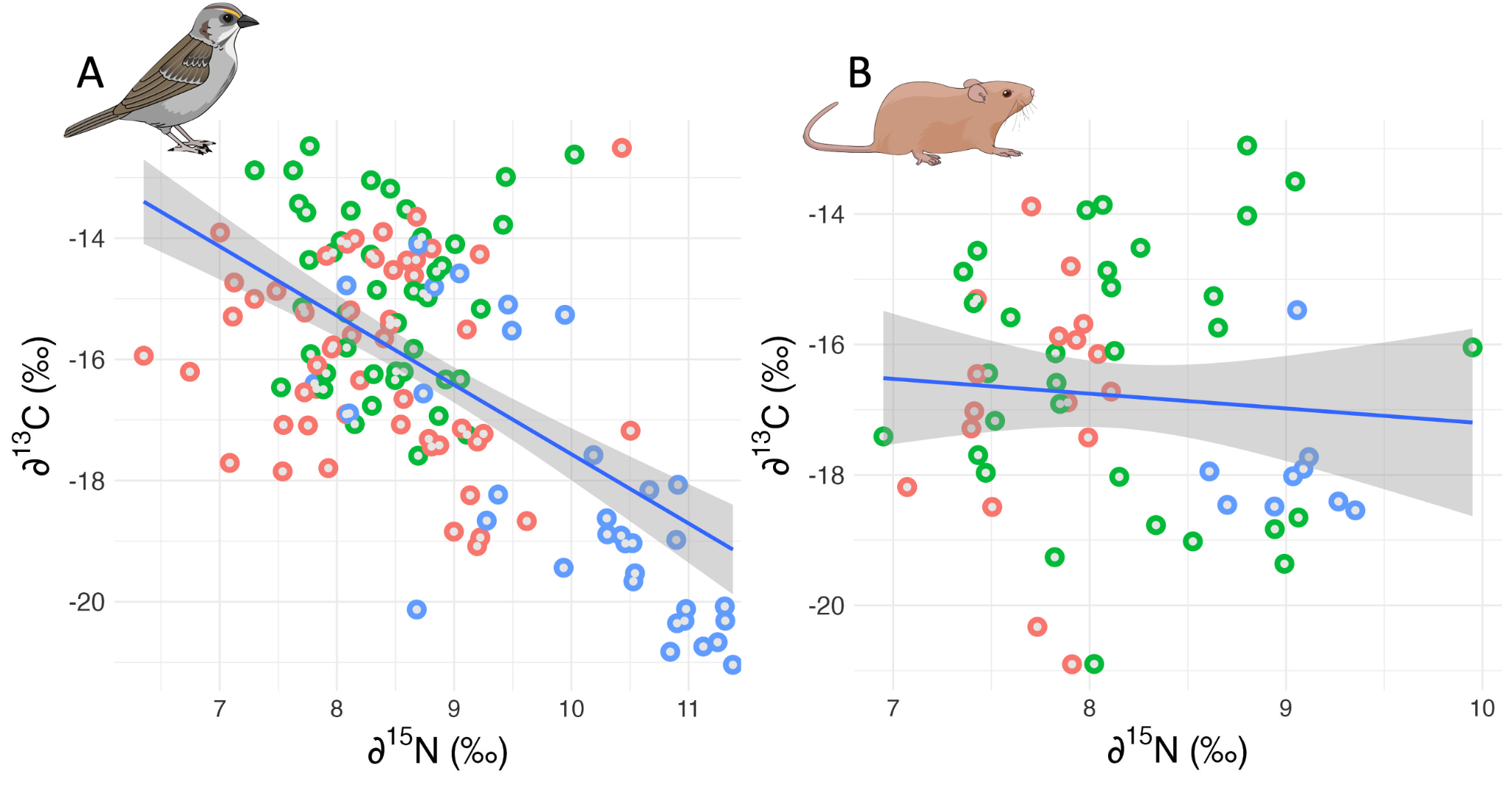


Figure S2. Covariation between nitrogen (∂^15^N) and carbon (∂^13^C) stable isotopes values in (A) Seaside Sparrows and (B) Marsh Rice Rats in sites with different oiling histories (control = green; oiled = red; east of the Mississippi River = blue).
